# Designing machine learning workflows with an application to topological data analysis

**DOI:** 10.1371/journal.pone.0225577

**Published:** 2019-12-02

**Authors:** Eric Cawi, Patricio S. La Rosa, Arye Nehorai

**Affiliations:** 1 Preston M. Green Department of Electrical and Systems Engineering, Washington University in St. Louis, St. Louis, MO, United States of America; 2 Global IT Analytics, Crop Science Division, Bayer Company, Saint Louis, MO, United States of America; Politechnika Krakowska im Tadeusza Kosciuszki, POLAND

## Abstract

In this paper we define the concept of the *Machine Learning Morphism (MLM)* as a fundamental building block to express operations performed in machine learning such as data preprocessing, feature extraction, and model training. Inspired by statistical learning, MLMs are morphisms whose parameters are minimized via a risk function. We explore operations such as composition of MLMs and when sets of MLMs form a vector space. These operations are used to build a machine learning workflow from data preprocessing to final task completion. We examine the Mapper Algorithm from Topological Data Analysis as an MLM, and build several workflows for binary classification incorporating Mapper on Hospital Readmissions and Credit Evaluation datasets. The advantage of this framework lies in the ability to easily build, organize, and compare multiple workflows, and allows joint optimization of parameters across multiple steps in an application.

## 1 Introduction

In this paper, we leverage the concept of morphisms to construct a general structure that represents all type of data operations including data preparation, feature extraction, and mapping to outcome of interest. We call these fundamental building blocks *Machine Learning Morphisms* (MLMs). We describe an entire workflow, from data preparation to final task evaluation, in a single equation featuring a composition of MLMs. This allows data scientists to easily keep track of the workflow, and provides a modular framework for workflow construction and comparison. Our framework was inspired by the minimization framework in statistical learning theory [1] and the composition of functions used by artificial neural networks [2]. The concept of morphisms was referenced in [3] as a mapping between human behavior and machines. However, to the best of our knowledge, expressing a sequence of data operations as a single composition of morphims is a novel construction. This construction allows us to easily build and compare workflows across a data set (or collection of datasets), clearly present and interpret the end results, and gives interesting possibilities for joint optimization over the paramters of every step in a workflow.

To illustrate this framework, we examine the specific case of binary classification, with imbalanced classes and hybrid continuous-categorical data. We examine the use of the Topological Data Analysis (TDA) algorithm Mapper [4] as an MLM, and build a workflow using Mapper to train an ensemble of classifiers. We compare workflows using Mapper to other workflows in numerical experiments on two real world datasets. Further, Mapper is generally used for feature selection and qualitative visual analysis, so our approach represents an innovative use of the algorithm.

The rest of this paper is organized as follows. Section II introduces the MLM, and defines several operations. In Section IV, we build and compare several workflows on two datasets, and in Section V we present some final thoughts and directions for future work. Table 1 gives the general form for notation used for spaces, functions, and other structures used in the paper.

**Table 1:** Notation for sets, spaces, functions, etc used throughout the paper.

Notation	Meaning	Example
Script Capital	Space	S
Bold, Non-Italic	Set	**X**
Bold, Italic, Lower Case, Index	Array	**x**, **x**_1_, **y**
Capital Italic	Function	*P*(⋅)
Italic, lower case	Scalar	*n*

## 2 Mathematical nomenclature for machine learning workflows

### 2.1 Statistical learning overview

To provide motivation and set up the class of problems we are interested in, we present a basic overview of the supervised statistical learning problem. In this problem, measurements, observations, or *data* are gathered with the goal of constructing a mathematical relationship between the data and an outcome of interest. Miescke and Liese [5] define the *sample space* as a pair (S,U) where S=X×Y is a space where the data X and outcomes Y can be observed, and U is a *σ*−algebra containing all subsets of S relevant to the problem. Elements s∈S are the *realizations* of a random variable *S* for some underlying probability space (**Ω**, **F**, *P*_**Ω**_). Here *P*_**Ω**_ is a probability measure on a set **Ω** and *σ*-algebra **F**, and the mapping S:Ω→S is measurable [5]. Let the set of *n* realizations of S be denoted as S∈S×S×⋯×S, where we call S×S×⋯×S the *statistical space*. The elements of **S** are *data points* or *samples*.

We will denote the realizations of the outcome of interest as $Y\in Y\times Y\times \cdots \times Y=Y^{n}$, and we will call the realizations of the features as $X\in X\times X\times \cdots \times X=X^{n}$. In [1], Vapnik et al. give three criterion for a supervised learning problem. The first is the underlying distribution of the features, *P*(*X* = **x**). Next is the unknown conditional distribution *P*(*Y* = **y**|*X* = **x**). Finally, given a parameter space Θ with elements **p**, denote the function

$$F:X\to Y,\;\hat{y}=F\left(x;p\right)$$ (1)

used to approximate *P*(*Y* = **y**|*X* = **x**) as a *learning machine*. The parameters **p** are learned using a loss function

L:Y×F(X)→R,l=L(y,F(x;p)) (2)

as a measure of discrepancy between the predictions *F*(**x**;**p**) and the observed values **y**.

Then define the *expected risk function* as:

$$R:\Theta \to R,\;r=R\left(p;X,Y,F\right)=\int_{S}L\left(Y,F\left(X;p\right)\right)dP\left(X,Y\right)$$ (3)

where *P*(*X* = **x**, *Y* = **y**) = *P*(*Y* = **y**|*X* = **x**)*P*(*X* = **x**) is the potentially unknown probability measure on X and Y. This represents the expected value of the lost function across all realizations, and the optimal parameters are defined as:

$$p^{*}=\underset{p\in \Theta }{\text{arg}\;\text{min}}\;R\left(p;X,Y,F\right)$$ (4)

Statistical learning theory has a rich history, and the properties of learning machines are widely studied and researched [6]. For example, Linear Regression [7], Support Vector Machines (SVMs) [8], and Neural Networks [9] all use this optimization framework with different choices of learning machine, loss, and risk functions to train their parameters. In practice the parameters are learned by approximating Eq 3 with an *empirical risk function* [10] defined on the realizations:

$$\bar{R}:\Theta \to R,\;\bar{r}=\frac{1}{n}\sum_{i=1}^{n}L\left(y_{i},F\left(x_{i};p\right)\right)$$ (5)

and the optimal parameters are given by:

$${\bar{p}}^{*}=\underset{p\in \Theta }{\text{arg}\;\text{min}}\;\bar{R}\left(p;X,Y,F\right)$$ (6)

Empirical risk is a valid approximation for the expected risk when Eq 5 converges to Eq 3 in probability as *n* goes to ∞. The conditions for this convergence are discussed thoroughly in [10], [7], and [1].

Learning workflows consist of a sequence of operations acting on the realizations in the statistical space. We distinguish two types of operations in these workflows. The first set consists of processes such as standardization and sampling, which help guarantee the convergence of the empirical risk function to the continuous risk function defined in Eq 3. The second set of operations learn the parameters of the learning machine *F*, which can itself be defined as a composition of operations acting on the sample space. These operations are often separated into stages such as preprocessing, feature extraction/selection, model training, etc., and it can be difficult to understand what is actually happening to the original data or if one has built the best workflow for the task at hand.

The field of Automated Machine Learning seeks to address this challenge by algorithmically finding the best workflow. Packages such as TPOT [11] and auto-sklearn [12] create wrappers around the popular scikit-learn package in python, while Auto-WEKA [13] performs hyperparameter optimization over the WEKA platform [14]. TPOT constructs a graphical model of a workflow, and then uses a genetic algorithm to search the space of possible workflows [11], Auto-WEKA uses a bayesian framework to iteratively optimize model hyperparameters. Auto-sklearn builds off of Auto-WEKA, but also incorporates model performance on past datasets and creates ensemble models out 15 classifiers available in scikit-learn [12].

In our approach, we define a fundamental building block based off the idea of learning machines and risk minimization in statistical learning theory called the *Machine Learning Morphism (MLM)*, that can be used to systematically build and analyze each step in a machine learning application, and keep track of the data at each step in the process. Since some data operations do not necessarily fit the mathematical definition of functions, for example splitting the data into multiple training and hold-out sets for cross-validation, we use morphisms as the building block rather than functions. This approach allows us to define a workflow as a composition of morphisms acting on the sample space, whose parameters are learned using a risk function acting on the statistical space.

### 2.2 The Machine Learning Morphism

**Definition 1**. Let:

- X be an input space, where X is part of a sample space {S,U} as defined in section 2.1,
- Y be an output space,
- F:X→Y a morphism from input to output spaces, with parameters **p** ∈ **Θ**, and
- *P*(**p**) a probability distribution on **p** representing prior knowledge of the parameters
- $\bar{R}:\Theta \to R$ an empirical risk function.

Then the *Machine Learning Morphism* ML:X→Y is defined as:

$$ML:X\to Y,\;\hat{y}=F\left(x;p^{*}\right)$$ (7)

where

$$p^{*}=\underset{p\in P}{\text{arg}\;\text{min}}\;\bar{R}\left(p;X,Y,F\left(\cdot ;p\right),P\left(p\right)\right)$$ (8)

and $X\subset X^{n}$ and $Y\subset Y^{n}$ are *n* realizations from the input and output spaces used to learn the parameters.

*Note 1*: The choice of a morphism with an associated empirical risk function and parameter prior represents a unique building block. We consider two MLMs with the same morphism *F* but different risk functions and/or priors to be different morphisms.

*Note 2*: For the purposes of simplicity we assume all of the risk functions used after this point are empirical risk functions acting on the training data rather than the expected risk function, and will refer to empirical risk functions as “risk functions”.

*Note 3*: We present linear regression, standardization, and other data operations as MLMs in Table 2.

**Table 2:** Common data operations expressed as MLMs.

Operation	Input Space X	Output Space Y	Parameters	Morphism	Empirical Risk Function
Data Encoding	Abstract Space X	$R^{m}$	embedding parameters	Injective map: $F:X↪R^{m}$	trivial (one—hot encoding) or cost function, e.g. from [20]
PCA	$R^{m}$	$R^{c}$	c∈N $A\in R^{m\times c}$	**x** **A**	$∥X-{\text{XA}}^{T}{∥}_{F}^{2}$ Such that: **AA**^*T*^ = **I**
Linear Regression	$R^{m}$	R	$p\in R^{m}$	**x** ⋅ **p**	${∥Y-{Xp∥}_{2}}^{2}$
Logistic Regression	$R^{m}$	[0, 1]	$p\in R^{m}$	$F\left(x.p\right)=\frac{1}{1+\text{exp}(-x\cdot p)}$	Maximum Likelihood $\prod_{i=1}^{N}F{\left(x_{i};p\right)}^{y_{i}}{\left(1-F\left(x_{i};p\right)\right)}^{1-y_{i}}$
SVM	$R^{m}$	{−1, 1}	$\left(w,b\right)\in \left(R^{m},R^{m}\right)$ slack variables $s\in R^{m}$, c∈R	**w** ⋅ **x** − **b**	∥**w**∥_2_ + *c*∥**s**∥_2_
Decision Tree	Set **X**	{*y*_1_, *y*_2_, …, *y*_*k*_} for finite *k*	splitting criterion	Tree	Gini Impurity $\sum_{j=1}^{N}1-\sum_{i=0}^{1}P{\left(Y_{j}=i|X_{j}=x_{j}\right)}^{2}$ Information Gain see [21]
Standardization	$R^{m}$	$R^{m}$	$\left(c,s\right)\in \left(R^{m},R^{m}\right)$	(**x** − **c**)diag(**s**)^−^1	*KL* Divergence, Eq 13
Adaboost	$R^{m}$	R	parameters associated with weak learners	$\sum_{i=1}^{k}F_{i}\left(x\right)$	Exponential Loss [22]: $\sum_{i=1}^{N}\text{exp}\left(-y_{i}\sum_{j=1}^{k}F_{j}\left(x_{i}\right)\right)$
Neural Networks	$R^{m}$	R	Weights in $R^{w}$	*F* = *F*_*k*_(*F*_*k*−1_(*F*_*k*−2_(…*F*_1_(**x**)))))	Loss functions, examples include Mean Squared Error: ∥**Y** − *F*(**X**)∥^2^, Cross Entropy: $\frac{-1}{N}\sum_{i=1}^{N}y_{i}\text{log}\left(F\left(x_{i}\right)\right)-$ (1 − *y*_*i*_)log(1 − *F*(**x**_*i*_)) [23]
Model Evaluation	Collection $V_{Y}^{k}$	R or $R^{k}$	Evaluation parameters Test/validation set	Performance Metric e.g. Accuracy, Sensitivity, etc.	Complexity Criterion or other objective e.g. Aikeke Information Criterion

The Machine Learning Morphism consists of the morphism *F*, whose parameters have been optimized over an empricial risk function $\bar{R}$ on the set of realizations from the statistical space. The risk function also performs any necessary operations on the data in the statistical space to ensure convergence in probability to the expected value risk function in Eq 3. One example of this is applying sampling techniques such as oversampling, undersampling, SMOTE [15], or ROSE [16] to the training data in a classification task. This helps “learn” a better representation of the training data conditioned on each class.

The morphism *F* represents an operation acting on data, such as data standardization, feature extraction, or regression, In supervised tasks, we assume that the realizations of Y are matched to corresponding realizations of X. In an unsupervised task or if training outputs are unknown (for example, k-means clustering), the realizations **Y** = ∅ are empty.

Consider the example of least squares linear regression. The input space X is $R^{m}$. Denote $X\in R^{n\times m}$ as the matrix made from *n* realizations of X. The output space Y is R and denote the *n*-dimensional vector of all output realizations as **Y**. We assume no prior knowledge on the parameters of *F*, so we use the improper prior $P\left(p\right)=1\forall p\in R^{m}$. The morphism *F* is defined as:

F(x;p)=x·p (9)

is the dot product of **x** and $p\in R^{M}$, and the empirical risk function *R* is the sum of squared error across all of the datapoints:

$$\bar{R}\left(p;X,Y,F\left(\cdot ;p\right),P\left(p\right)\right)=∥Y-{Xp∥}_{2}^{2}$$ (10)

where ∥⋅∥_2_ is the standard 2-norm for real-valued vectors. The Machine Learning Morphism is

$$ML\left(x\right)=x\cdot p^{*}$$ (11)

where $p^{*}=\text{arg}\;\text{min}∥Y-{Xp∥}_{2}^{2}={\left(X^{T}X\right)}^{-1}X^{T}Y$ is the optimal least squares solution, and $X^{T}\in R^{m\times n}$ is the transpose of **X**.

Continuing the example of linear regression, many times the data matrix **X** consists of shifted and scaled columns. The process of shifting and scaling is an MLM. The input space is $R^{m}$, and the output space is $R^{m}$. The parameter **p** is a vector containing shifting constants $c\in R^{m}$ and scaling constants $s\in R^{m}$, and again *P* is the non-informative prior. The morphism

$$F\left(x;p\right)=\left(x-c\right)\text{diag}{\left(s\right)}^{-1}$$ (12)

where diag(**s**) is the diagonal matrix whose main diagonal is **s**. This morphism shifts and scales each element of *x*. The risk function depends on the type of scaling implemented. For example, the goal of standardization is to transform data into standard Gaussian random variables to ensure that each element of a data point lies on the same scale for comparison, statistical tests, or model training later on in the workflow. For standardization, one choice of empirical risk function is

$$\bar{R}\left(p;X,Y,F\left(\cdot ;p\right),P\left(p\right)\right)=KL\left(P\left(\left(X-1^{m}⊗c^{T}\right)\text{diag}{\left(s\right)}^{-1}\right),N\left(0,I_{n}\right)\right)$$ (13)

where *KL*(⋅) is the *KL* divergence [17] between the distribution of their shifted and scaled data and the standard multivariate normal distribution, **I**_*n*_ is an *n* × *n* identity matrix, and ⊗ is the Kronecker product. Let $\bar{x}$ be the vector of column means of **X**, and $\tilde{x}$ be the vector of standard deviations of each columns of **X**. If the data follow a multivariate normal distribution, then the optimal parameters in this case are $c=\bar{x}$ and $s=\tilde{x}$.

Because we specify a prior distribution on parameters in Definition 1, we can express Bayesian operations as MLMs. Consider the Naive Bayes classifier [18] with input space is a set X. Similarly, the output space is the set $Y=\{y_{1},...,y_{k}\}$ for *k* < ∞. Assuming conditional independence between the input data, the morphism chooses the class which maximizes the posterior distribution, $\underset{i\in \{1,...,k\}}{\text{arg}\;\text{max}}\;P\left(Y=y_{i}|X=x;p^{*}\right)$, and the empirical risk function is 0-1 loss [18]. The parameter prior *P*(**p**) is a distribution on the parameters of the likelihood *P*(*X* = **x**|*Y* = *y*; **p**) and prior *P*(*y*; **p**).

Now that we shown examples of three MLMs, it is natural to investigate how they relate to one another. Property 1 establishes that for certain risk functions, learning the parameters for one MLM can be decomposed into learning the parameters of two MLM’s with lower dimensional parameter spaces and potentially lower dimensional input spaces.

**Property 1**. First define two separate MLMs with the same output space: ${ML}_{1}$ with input space $X_{1}$, output space Y, parameter prior *P*(**p**_1_), morphism *F*_1_, risk function ${\bar{R}}_{1}$; and $ML_{2}$ with input space $X_{2}$, output space Y, parameter prior *P*(**p**_2_), morphism *F*_2_, risk function ${\bar{R}}_{2}$. Let the Θ_1_ and Θ_2_ represent the respective parameter spaces for **p**_1_ and **p**_2_.

Let ⊕ represent a closed operation defined on Y, $X_{3}=X_{1}\cup X_{2}$ be an input space, and **p**_3_ = (**p**_1_, **p**_2_) ∈ Θ_1_ × Θ_2_ be a parameter vector. Define $ML_{3}$ as an MLM with input space $X_{3}$, output space Y, parameter prior *P*(**p**_3_), morphism *F*_3_(**x**_3_; **p**_3_) = *F*_1_(**x**_1_; **p**_1_)⊕*F*_2_(**x**_2_; **p**_2_), where $x_{1}\in X_{1}$, $x_{2}\in X_{2}$, and **x**_1_∪**x**_2_ = **x**_3_. Denote the risk function for $ML_{3}$ as ${\bar{R}}_{3}\left(p_{3};X_{3},Y,F\left(\cdot ;p_{3}\right),P\left(p_{3}\right)\right)$. Note that the parameter prior *P*(**p**_3_) is the joint distribution of **p**_1_ and **p**_2_.

If the risk function ${\bar{R}}_{3}$ has the form $a{\bar{R}}_{1}\left(p_{1};X_{1},Y,F_{1}\left(\cdot ;p_{1}\right),P\left(p_{1}\right)\right)+b{\bar{R}}_{2}\left(p_{2};X_{2},Y,F_{2}\left(\cdot ;p_{2}\right),P\left(p_{2}\right)\right)$, where $a,b\in R^{+}$, then $ML_{3}\left(x_{3}\right)$ is equivalent to ${ML}_{1}\left(x_{1}\right)⊕ML_{2}\left(x_{2}\right)$.

*Proof*: In appendix.

Examples of closed operations between MLMs when Y=R include addition, multiplication of two MLMs (for example multiplying probability density functions), and division. If the output space is binary, then Boolean logic operations are valid.

In the example of linear regression, the least squares risk function can be decomposed into two lower dimensional least squares risk functions if the training realizations **X** form an orthogonal matrix. This could be a property of the feature space or the result of applying a transformation such as principal component analysis. To see this decomposition, let $ML_{3}$ be the MLM representing linear regression trained over **X**, with regression coefficients **p**. Split the parameters $p=[\begin{array}{c} p_{1}\\ p_{2} \end{array}]$ where $p_{1}\in R^{c}$ and $p_{2}\in R^{m-c}$ and the training matrix into **X** = [**X**_1_**X**_2_] where *X*_1_ is the first *c* columns of **X**, and **X**_2_ contains the remaining columns. Let ${ML}_{1}$ and *ML*_2_ represent linear regressions trained on **X**_1_ and **X**_2_ respectively using empirical risk functions ${\bar{R}}_{i}=∥Y-X_{i}p_{i}{∥}_{2}^{2}-\frac{1}{2}{∥Y∥}_{2}^{2}$ for *i* = 1, 2. For $ML_{3}$, the risk function is:

$${\bar{R}}_{3}=∥Y-{Xp∥}_{2}^{2}={\left(Y-Xp\right)}^{T}\left(Y-Xp\right)=$$ (14)

$$-\frac{1}{2}{∥Y∥}_{2}^{2}+∥Y-X_{1}p_{1}{∥}_{2}^{2}-\frac{1}{2}{∥Y∥}_{2}^{2}+∥Y-X_{2}p_{2}{∥}_{2}^{2}={\bar{R}}_{1}+{\bar{R}}_{2}$$ (15)

which clearly satisfies Property 1. In the derivation (provided in the appendix) we leverage the fact that $X_{1}^{T}X_{2}$ and other cross terms form a zero matrix due to the orthogonality of **X**. In this case each regression coefficient can be found by solving it’s own independent one dimensional least squares problem, which could be easily parallelized for a very fast regression.

If Y is a vector space, then ensemble models featuring linear combinations of independently trained MLM’s can be decomposed into problems with lower dimensional parameter spaces. For example in random forests each tree is independently trained (common risk functions include Gini Impurity or Information gain) on random and possibly overlapping subsets of the feature space using the random subspace method [19]. The parameters of each tree are the splitting criterion, and the output of a random forest is the average response of all trees in the forest, which is used either as a majority vote or class probability. Because the trees are trained independently of each other, we can define a risk function for the random forest as the sum of the Gini Impurity in each tree and use Property 1 to decompose the forest into its individual trees.

We are also interested in sequential operations using MLMs, so we need a notion of composition. For example, we wish to first standardize our data, and then perform linear regression.

**Definition 2**. Let ${ML}_{1}$ and $ML_{2}$ be two MLMs such that the output space of ${ML}_{1}$ is the same as the input space of $ML_{2}$. We define the *composition* $ML_{3}=ML_{2}\circ {ML}_{1}$ as a MLM with structure:

$$\text{Input}\;\text{Space}:X_{1}$$ (16)

$$\text{Output}\;\text{Space}:Y_{2}$$ (17)

$$\text{Parameter}\;\text{Prior}:P\left(p\right)≔P\left(p_{1},p_{2}\right)$$ (18)

$$\text{Learning}\;\text{Morphism}:F\left(x,p\right)=F_{2}\left(F_{1}\left(x;p_{1}\right);p_{2}\right)$$ (19)

$$\text{Empricial}\;\text{Risk}\;\text{Function}:{\bar{R}}_{2}\left(p_{2};X,Y,F\left(\cdot ;p_{1}\right),P\left(p\right)\right)$$ (20)

and the learned parameters are given by

$$p^{*}=\underset{\left(p_{1},p_{2}\right)}{\text{arg}\;\text{min}}\;{\bar{R}}_{2}\left(p_{2};X,Y,F\left(\cdot ;p_{1}\right),P\left(p_{1},p_{2}\right)\right)$$ (21)

The output morphisms is a composition of *F*_1_ and *F*_2_ follows the general form for composition of morphisms, and reflects the sequence of transformations on data in a machine learning application. The risk function used in this definition optimizes the task over every available parameter. This was motivated by the idea that this risk function is oriented to the task or machine learning goal, so there is an opportunity to improve task performance by jointly optimizing the parameters.

In our example of standardization (${ML}_{1}$) followed by linear regression ($ML_{2}$), $ML_{3}=ML_{2}\circ {ML}_{1}$ has structure:

$$X=R^{m}$$ (22)

Y=R (23)

$$F\left(x,\left(p,c,s\right)\right)=\left(x-c\right)\text{diag}{\left(s\right)}^{-1}\cdot p$$ (24)

$$\bar{R}\left(p;X,Y,F\left(\cdot ,\left(p,c,s\right)\right),P\left(p,c,s\right)\right)=$$ (25)

$$∥Y-\left(X-1^{m}⊗c^{T}\right)\text{diag}{\left(s\right)}^{-1}p{∥}_{2}^{2}$$

In linear regression, standardization is extremely common, and MLMs such as PCA (shown in section 3) require standardized input data. However, in some applications it may be of interest to give more weight to one column than another, and exploring non-standard or non-linear scalings may be an interesting future application.

### 2.3 Collections of morphisms

In machine learning, we are often interested in ensembles of models, evaluating model performance, and model selection. To incorporate these tasks into the MLM framework, we need a notion of multiple MLMs gathered together.

**Definition 3**. Given MLMs ${ML}_{1}$, $ML_{2}$, …,$ML_{k}$, then the *collection* $C_{ML}$ of MLMs is the set:

$$C_{ML}≔{\left\{ML_{i}\right\}}_{i=1}^{k}$$ (26)

We call the number of morphisms, *k*, the dimension of $C_{ML}$.

In the numerical experiments presented in Section IV, we use the idea of collections for model selection and evaluation. Once data has been collected, it is standard practice in machine learning to split the data into a *Training Set* used to develop a workflow and choose optimal parameters, a *Validation Set* used to assess and tune model performance during development, and a *Testing Set* used to assess performance after development is completed [24]. Evaluating the performance of a model is a MLM, which we will denote $ML_{eval}$.

The input space is the set of all collections of *k*-dimensional MLMs with output space Y, which we will denote $V_{Y}^{k}$. The output space (often R or $R^{k}$) represents the performance of the model(s) under consideration.

There are two major sets of parameters. The first are any parameters **p**_1_ required by the morphisms in the collection, which have prior *P*(**p**_1_). The second are the parameters of the evaluation function, **p**_**2**_, which have prior *P*(**p**_2_). Assuming these parameters are indepentent, the final parameter prior is *P*(**p**_**1**_)*P*(**p**_2_).

The morphism evaluates the performance of the collection of MLMs on a new set of realizations. Examples include sensitivity, the Reciever Operating Characteristic (ROC) [25], or the Aikeke Information Criterion (AIC) [26]. A model selection morphism chooses the MLM in a collection that maximizes the chosen performance measure. The risk function performs whatever optimization is required by the performance measure, such as complexity criterion.

### 2.4 Machine learning workflow

Now that we have established operations and composition on MLMs, we define the *Machine Learning Workflow* (MLW or “workflow”).

**Definition 4**. A *Machine Learning Workflow* is a finite composition of *k* MLMs $M=ML_{k}\circ ML_{k-1}\circ \ldots \circ ML_{2}\circ {ML}_{1}$, with initial input space X and final output space Y.

This definition is inspired by the formulation of artificial neural networks as compositions of functions. Instead of sigmoids, our composition represents different steps in a machine learning application such as data preprocessing, feature extraction, and model training. A workflow can contain any number of MLMs, and each MLM could possibly be broken down into more MLMs. This representation allows us to present an application with a single master equation, which keeps track of the parameter space, and optimizes over a single risk function. This has advantages in both presentation and organization. If we are evaluating several workflows over the same outcome space (for example, models created for the Netflix Competition), we can use the MLM framework to create an ontology of workflows for evaluation and comparison.

### 2.5 Binary classification use case

We are specifically interested in the case when data points x∈X are sets composed of both real-valued continuous variables and categorical variables, the output space is Y={0,1} (binary classification). Further, we are interested in the case when there is a class imbalance, e.g. significantly more observed samples where *y* = 0 than *y* = 1. We were developing a machine learning model to identify hospital patients at high risk of 30-day unplanned readmission, which occurs when patients come back to the hospital within 30 days of discharge. We developed this framework to better keep track of how the data was manipulated, and discovered a potentially useful application of the Topological Data Analysis algorithm Mapper as an MLM. The rest of this paper will describe workflows we developed in terms of the MLM framework.

## 3 TDA Mapper as a MLM

In this section we present a brief overview of the TDA Mapper algorithm, show that it fits into the MLM framework, and build some example workflows.

### 3.1 Mapper algorithm

Topological Data Analysis assumes that the space X can be endowed with a collection of subsets O. Elements o∈O satisfy $\underset{i}{\cup }o_{i}\in O$ and $\underset{i=1}{\overset{n<\infty }{\cap }}o_{i}\in O$, and are called open sets. Then the ordered pair {X,O}, forms a *Topological Space*. TDA builds topological spaces on top of data points, and evaluates the shape of the computed spaces [27]. The critical features are closed loops in various dimensions, which are invariant to rotation or multiplicative scaling. When X is also endowed with a probability space, the topological features are also endowed with a probability space [28], and can be used in machine learning. TDA has been used as a novel visualization tool in bio-medical applications [29] [30], text mining [31], and remote sensing [32].

One visualization tool developed for TDA at Stanford is the Mapper Algorithm [4]. It creates a graphical representation of the data that keeps an equivalent topological structure, and has been used in a wide variety of applications [33] [34]. Mapper is usually used as a method for clustering and visualization. Interesting clusters or patterns are used as a feature selection method to reduce the dimensionality of data before training learning models.

To construct the Mapper graph, first define a filtration function A:X→R (*note*: it isn’t necessary for the range to be R, but we’re using it here for simplicity). Then define an equivalence relation ∼_*A*_ such that **x**_1_ ∼_*A*_ **x**_2_ whenever *A*(**x**_1_) = *A*(**x**_2_), which collapses every level set of *A* to a single point. The *Reeb Graph* is the quotient space of X under the relation ∼_*A*_. Mathematically, the Mapper algorithm computationally approximates the Reeb Graph by computing the nerve of a refined pullback of an open cover, O⊂O, of A(X). Practically, Mapper assigns datapoints to **O**, and then performs clustering within each member of the open cover. Then it creates graph *G* with a set of nodes **N**_*i*_ ∈ **N** representing the clusters, and a set of edges **E** where an edge *e*_*ij*_ means that two clusters have non-empty intersection. It has been proven to converge exactly to the Reeb graph if **O** is refined enough [35]. The full algorithm is described in [4], and rough pseudocode of computational algorithm is detailed in Algorithm 1.

In topology, an *abstract simplicial complex* is a family of non-empty finite sets that is closed when taking non-empty subsets. One of the main ideas of TDA is to create abstract simplicial complexes from sets of data [36]. The pullback operator on an open cover has a more complicated definition that is out of the scope of this paper, but the *nerve* of an open cover is a representation of the open cover as an abstract simplicial complex. The Mapper algorithm computes the nerve of the O using the procedure in Algorithm 1, and the result is a graph showing the “shape” of the data [4].

**Algorithm 1** Description of the TDA Mapper Algorithm by Singh, Memoli, and Carlsson et al.

**Input**:  • Data **X**, distance metric *D* on X, filtration function A:X→R

   • Number of intervals *k*, number of bins when clustering *b*, percent overlap *o*

**Output**: Graph *G*, nodes *n*_*i*_ ∈ **N**, edges *e*_*ij*_ ∈ **E**

1: **N** ← ∅

2: **E** ← ∅

3: **Compute Y** = *A*(**X**)

4: Generate an open cover of **Y** with *k* open intervals ${\left\{I_{j}\right\}}_{j=1}^{k}$, with area *a*_*I*_, such that **I**_*j*_ ∩ **I**_*j*+1_ ≠ ∅ and the area of each intersection is o*a_*I*_

5: **for** *j* = 1 to *k* **do**

6:  Perform clustering such as k-means, using *b* clusters, on **x** ∈ **X** ∩ *A*^−1^(**I**_*j*_)

7:  append each cluster **N**_*i*_, for *i* = 1, 2, 3… to **N**

8: **end for**

9: **for** *i*, *j* ∈ **N** **do**

10:  **if** **N**_*i*_ ∩ **N**_*j*_ ≠ ∅ **then**

11:   Append edge *e*_*ij*_ to **E**

12:  **end if**

13: **end for**

14: **return N**, **E**

### 3.2 Machine learning workflows with Mapper

The set of nodes, **N** = {**N**_1_, …, **N**_*w*_}, output by Mapper, represents a cover, ${\left\{X_{i}\right\}}_{i=1}^{w}$ of **X**. Taking Mapper as a morphism from X to the set of all covers of X, the Mapper algorithm is an MLM with structure:

- Input space is the topological space (X,O)
- Output space is the set of all open covers of X
- A Parameter Prior over the parameters in algorithm 1 *P*(*D*, *A*, *k*, *b*, *o*), representing prior knowledge or choices of the proper distance metric, filtration function, etc.
- Morphism: Nerve of the refined pullback of an open cover, **O**, of A(X)
- Risk function: Graph Edit Distance [37]

Because Mapper approximates the Reeb Graph, we use the graph edit distance (GED) [37] between Mapper and the “true” Reeb Graph as the risk function for the Mapper MLM. The graph edit distance between graphs *G*_1_ and *G*_2_ summing up the cost of the operations necessary to transform *G*_1_ into *G*_2_. These operations commonly include adding/deleting edges, nodes, and changing the labels of nodes. Formally if **B** = [*B*_1_, *B*_2_, …, *B*_*k*_] contains the graph operations necessary to transform *G*_1_ into *G*_2_ and $C:B\to R^{+}$ is a cost function, then the graph edit distance is:

$$\bar{R}=GED\left(G_{1},G_{2}\right)=\sum_{i=1}^{k}C\left(B_{i}\right)$$ (27)

In Section IV, we use grid search to search through the parameters of Mapper. To bring this MLM closer to the realm of statistical learning theory, future work could extend the statistical analysis from [35] to define an computationally tractable loss function and more informative parameter prior. However, in the context of the larger workflow, the choice of risk is less relevant because the parameters are optimized over the final risk function.

We build an example machine learning workflow with Mapper and logistic regression as follows:

- The input space is X, which has training realizations **X**_*TR*_, validation realizations **X**_*V*_, and testing realizations **X**_*TS*_.
- The output space is Y={0,1}.
- $ML_{0}$: Dummy coding the original data matrix, embeds the data into $R^{m}$.
- ${ML}_{1}$: Mapper MLM on trained on realizations **X**_*TR*_:
  - If the first principal component is the filtration function *a*, then this MLM features a composition with the PCA MLM.
  - For computational reasons down the line, we remove vertices with less than 40 data points, so the output is not a total cover of X. When the first principal component is used this seems to have the effect of removing outliers with high/low PCA scores.
  - ${ML}_{1}$ outputs a set of spaces ${\left\{X_{i}\right\}}_{i=1}^{k}$, with training realizations separated into each group $\left\{X_{TR,i}\subset X_{i}\right\}$
- $ML_{2}=\sum_{i=1}^{w}C_{i}\left(x\right){ML}_{2}^{i}$, where each ${ML}_{2}^{i}$ has structure:
  - Input Space: $R_{m}$,
  - Output Space: Y=[0,1], representing the class probability *P*(*y* = 1),
  - Morphism: Composition of:
    - Feature extraction, e.g. PCA,
    - A learning machines, e.g. Logistic Regression trained on **X**_*TR*,*i*_
  - Parameter Prior: Gaussian priors on the regression coefficients for each node, uniform priors on the parameters of the Mapper MLM.
  - Risk function: Maximum likelihood, combined with sampling to address class imbalance, e.g. oversampling, undersampling, ROSE [38], or SMOTE [15]. Additionally, model hyperparameters can be selected with cross validation.
  - $C_{i}:X\to R$ is a weighting function depending on where points lie in the input space, related to which nodes of the Mapper graph are “active” for a given data point.
- $ML_{3}$: A decision threshold with
  - Input space: X=[0,1]
  - Output space: Y={0,1}
  - Morphism:
    $$\left\{ \begin{array}{cc} y=1 & \text{if}\;x\geq T\\ y=0 & \text{else} \end{array}\right.$$ (28)
  - Parameter Prior: prior information of threshold *T* ∈ [0, 1]
  - Risk Function: Method to choose probability threshold, e.g. choosing an optimal threshold of a ROC curve over cross validation sets.

The full workflow is:

$$M:X\to \left\{0,1\right\}=ML_{3}\circ ML_{2}\circ {ML}_{1}\circ ML_{0}$$ (29)

**Fig 1:**
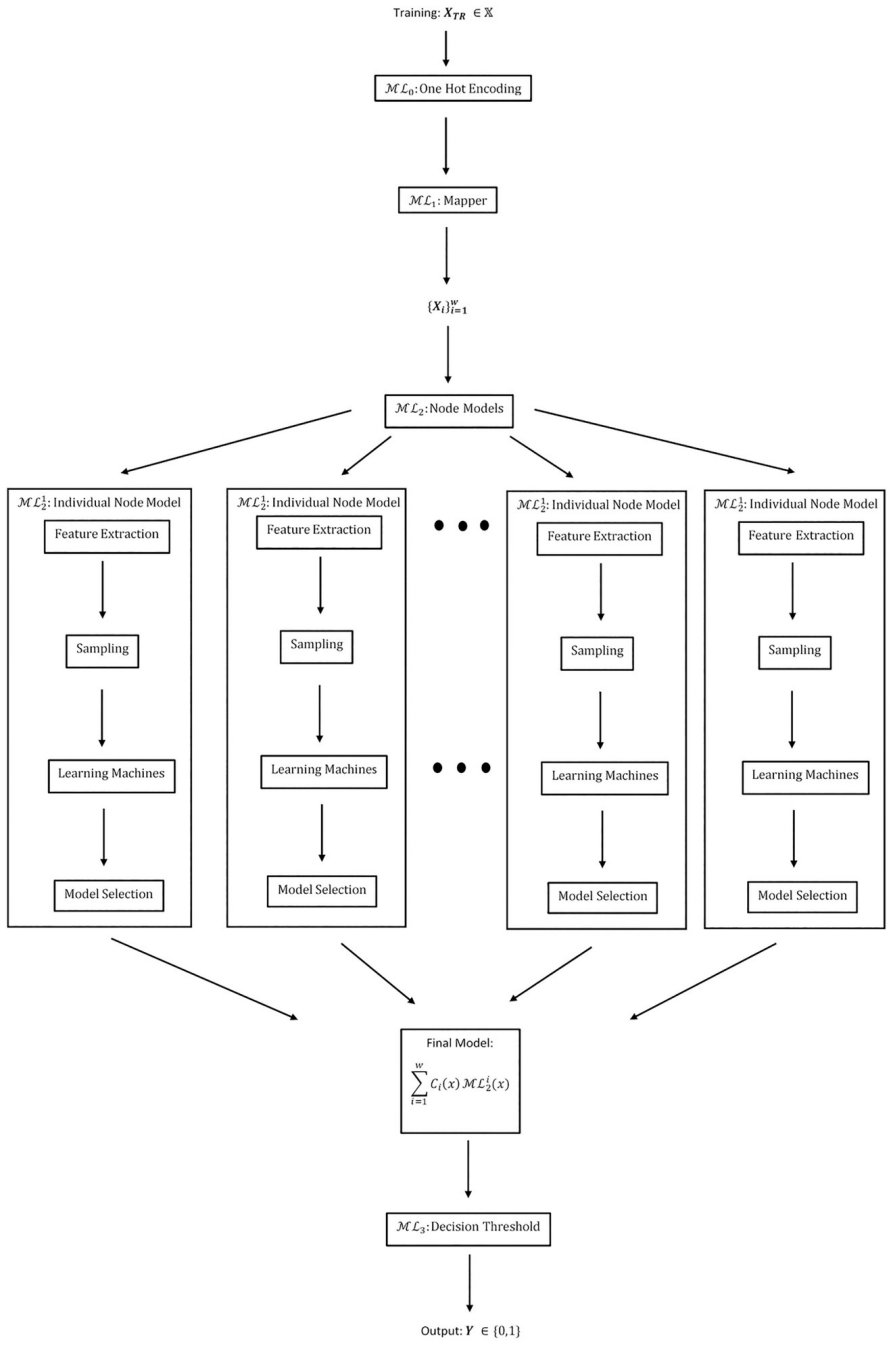
Block diagram of Eq 29, showing how a workflow is created for each node. The first step is one-hot encoding the data to embed it into $R^{M}$. The next step computes the Mapper graph of the data. Then models are trained on each node, and summed. Finally, a decision function outputs the final class prediction.

This MLW creates and optimizes a separate workflow ${ML}_{2}^{i}$ for each node created by the Mapper graph. Then, we leverage Property 1 and the fact that each workflow outputs class probabilities to create a weighted ensemble of workflows. Fig 1 shows a block diagram represenation of the full workflow using Mapper. Final model evaluation uses M as an input to an evaluation MLM using realizations from the test set **X**_*TS*_. The weights *C*_*i*_(*x*) in $ML_{2}$ represent an interesting choice. Intuitively, weights should be non-zero only when a point lies in $X_{i}$, so only a portion of the models are “active” for a given point. Because they are summing elements of a probability space, $\sum_{i=1}^{w}C_{i}\left(x\right)=1$ for all **x**. Options for the weighting parameters include:

- Assign a weight of 1 to the “closest” node and 0 to all others.
- Assign equal weight to all nodes to which the point belongs, and 0 to all others.
- Assign weight inversely proportional to the distance from the center of the interval assigned to that node.
- Assign weight proportional to the cross validation metrics of each model, i.e. models that perform better on the training data are assigned higher weights.
- Train weights within cross validation by defining a loss function based on the metric of interest.

The Mapper algorithm could be replaced with another clustering algorithm such as k-means, or any other mapping that chooses subsets of data. We chose the Mapper algorithm because the subsets it generates have some appealing properties. First, the points in one $X_{i}$ are all “close” in the sense of the filtration function, but may have a different internal structure than another node to which they are not connected. A column of **X**_*i*_ may be positively correlated with the outcome variable, but the same column of **X**_*j*_ may be negatively correlated. When considered in a model over the entire dataset, these correlations may “compete” with each other.

Furthermore, with the proper choice of a filtration function, some nodes may have a higher incidence of the outcome variable. In previous literature this was done in order to identify subsets with high minority prevalence for further study. By training a model only on that node, we reduce some of the class imbalance on that set, theoretically increasing model performance. Finally, many clustering methods do not produce overlapping clusters, but in [39], training classifiers on overlapping cluster was shown to improve performance. The Mapper algorithm allows for datapoints that fall into multiple nodes of the Mapper graph to contribute information to each node’s model.

To evaluate the workflow M, use the workflow as input to an MLM that evaluates classifier performance over new realizations from X (the testing set). In the next section we focus on ROC area under the curve (AUC), Sensitivity, and Specificity as different training metrics.

## 4 Numerical experiments

### 4.1 Comparisons

We built several versions of Eq 29 across two real world datasets. The workflow breakdown is as follows:

- Realizations: Always an 80/20 training/testing split.
- $ML_{0}$: Dummy coding performed using the default parameters from the caret package.
- ${ML}_{1}$: One of [Mapper, Identity (no transformations)]. For the Mapper graph, we used a uniform prior on the number of intervals *k* from [5–20], the percent overlap *o* from [20%–60%], and the number of bins when clustering from [5–30]. We fixed the filtration function *a* as the first principal component, and the distance metric *d* as the gower metric, which means we used a dirac delta as the prior for these parameters.
- $ML_{2}$: Node Models:
  - Feature Extraction: Used caret package in R [40] to perform one of: [no transformations, PCA]
  - Sampling: Used caret package in R to perform one of [no sampling, SMOTE, ROSE]
  - Learning Machines: Used caret package in R to train one of [Logistic Regression, SVM, Random Forests, AdaBoost [22]]
  - Cross Validation: Used caret package in R to generate 10-fold cross validation sets to tune model hyperparameters, such as the number of trees in the random forests.
  - Weighting functions *C*_*i*_: Points are assigned to nodes by computing the filtration function, assigning to appropriate intervals and then finding the closest cluster within each interval. Then weights were assigned to the one or two closest nodes. If two nodes are used, we use either equal weights or weights proportional to the ROC performance on the validation set.
- $ML_{3}:$ Decision threshold function as defined in Eq 28.

Mapper graphs used the first principal component as the filtration function, and the other parameters were tuned by grid search. Preliminary investigations with other filtration functions on patient record data revealed that the first PC seemed to yield the best classifier performance, so it was fixed as the filtration function for each experiment. These graphs tended not to find any loops or interesting topological structures when using that particular filtration function. However, there was usually a good spread with respect to the outcome variable where some nodes have a high minority class occurence and others have very low.

For the risk function, we optimized $ML_{2}$ independently of the whole process, via methods such as maximum likelihood (for Logistic Regression) using models from caret. The decision threshold in $ML_{3}$ and the parameters of the mapper algorithm were selected to optimize the average ROC AUC over the hold-out sets from the cross validation. This was done via grid search.

The results are grouped by the type of learning machine used in $ML_{2}$. Each workflow uses only one of these types, and experimenting with more involved model selection on different nodes **X**_*i*_ is an interesting direction of future work. Every workflow tested was run with 10 different training/testing splits, and the resulting performance measures were averaged. The workflows are named by Mapper/No Transformation, PCA (if applicatble), Sampling method (if applicable), node weights (if applicable).

### 4.2 Hospital readmissions data

The original dataset used in development of this project is from a set of 776 medicare/medicaid patients from Barnes Jewish Hospital in St. Louis, MO admitted between April 2015-April 2016. We seek to predict whether or not the patients will be readmitted into the hospital within 30 days after being discharged. The variables collected are: LACE Risk Score, presence of Diabetes, principal diagnoses from ICD9/10 codes, gender, ethnicity, zip code readmission rate, length of stay, age, and presence of a primary care provider. 134 (17.3%) of the patients were readmitted. In each run of the simulation, the data was divided into 621 training and 155 test patients. Some descriptions of the population under study are given in Table 3. Tables 4–7 show the classification results for multiple workflows, themed by the type of classifier, averaged over 10 runs. The chosen Mapper parameters were 10 intervals, 50% overlap, and 20 bins when clustering. Typical Mapper graphs had 10 nodes, each with 40-200 patients per node. We report the ROC AUC, Sensitivity, Specificity, and Accuracy for each model tested, but note that accuracy in this case is biased heavily towards the specificity score since negatives make up 83% of the patients.

**Table 3:** Descriptive table of patient data from Barnes Jewish Hospital.

	Total Cohort (N = 776)	Number Readmitted (Total Readmissions = 134)
Congestive Heart Failure (CHF)	378	71
Chronic Obstructive Pulmonary Disease (COPD)	88	9
Acute Myocardial Infarction (AMI)	198	31
Pneumonia (PNA)	113	23
Male	420	74
White	472	76
Has Diabetes	142	32
65-70 years old (y.o.)	209	41
70-75 y.o.	174	30
75-80 y.o.	135	18
85+ y.o.	258	45.
Discharged Home	296	50
Disch. to Skilled Nursing Facility	147	25
Disch. with Home Health	270	52
Low LACE Score (< 5)	30	3
Medium LACE Score (5-10)	231	27
High LACE Score (> 10)	515	104
Average Length of Stay (Days)	7.0	8.25

**Table 4:** Results for different workflows of logistic regression on hospital readmissions data, with standard deviations over n = 10 runs.

LR Workflow	ROC AUC	Sensitivity	Specificity	Accuracy
No Transformation	0.49 (0.023)	0.58 (0.031)	0.48 (0.021)	0.49 (0.022)
No Transformation, SMOTE	0.64 (0.033)	0.62 (0.029)	0.67 (0.039)	0.66 (0.037)
No Transformation, ROSE	0.53 (0.041)	0.54 (0.044)	0.51 (0.045)	0.52 (0.045
PCA	0.58 (0.017)	0.68 (0.022)	0.45 (0.029)	0.49 (0.028)
PCA, SMOTE	0.49 (0.037)	0.64(0.035)	0.44 (0.034)	0.47 (0.034)
PCA, ROSE	0.45 (0.061)	0.50 (0.059)	0.55(0.065)	0.54 (0.063)
Mapper, No Transformations	0.61 (0.048)	0.62 (0.052)	0.53 (0.049)	0.55 (0.050)
Mapper, No Transformations, SMOTE	0.67 (0.066)	0.60 (0.055)	0.60 (0.064)	0.60 (0.062)
Mapper, No Transformation, ROSE	0.62 (0.073)	0.69 (0.076)	0.59 (0.078)	0.61 (0.078)
Mapper, Node PCA	0.55 (0.065)	0.62 (0.058)	0.50 (0.059)	0.52 (0.058)
Mapper, Node PCA, SMOTE	0.69(0.071)	0.62 (0.069)	0.78 (0.065)	0.75 (0.066)
Mapper, Node PCA, ROSE	0.61 (0.084)	0.58 (0.082)	0.63 (0.087)	0.62 (0.086)

**Table 5:** Results for different workflows of SVMs for hospital readmissions data, with standard deviations over n = 10 runs.

SVM Workflow	ROC AUC	Sensitivity	Specificity	Accuracy
No Transformation	0.63 (0.033)	0.65 (0.037)	0.6 (0.035)	0.61 (0.036)
No Transformation, SMOTE	0.59 (0.042)	0.65 (0.040))	0.49 (0.046)	0.52 (0.044)
No Transformation, ROSE	0.55 (0.083)	0.80 (0.087)	0.44 (0.091)	0.50 (0.090)
PCA	0.64 (0.039)	0.69 (0.044)	0.58 (0.038)	0.60 (0.039)
PCA, SMOTE	0.61 (0.047)	0.58 (0.043)	0.58 (0.048)	0.58 (0.046)
PCA, ROSE	0.62 (0.058)	0.62 (0.049)	0.62 (0.054)	0.62 (0.053)
Mapper, No Transformations	0.53 (0.057)	0.58 (0.075)	0.48 (0.068)	0.49 (0.070)
Mapper, No Transformations, SMOTE	0.57 (0.079)	0.54 (0.072)	0.64 (0.076)	0.62 (0.075)
Mapper, No Transformation, ROSE	0.53 (0.086)	0.50 (0.081)	0.64 (0.088)	0.61 (0.086)
Mapper, Node PCA	0.50 (0.065)	0.62 (0.073)	0.49 (0.072)	0.51 (0.072)
Mapper, Node PCA, SMOTE	0.61 (0.077)	0.73 (0.083)	0.53 (0.089)	0.56 (0.088)
Mapper, Node PCA, ROSE	0.67 (0.092)	0.77 (0.095)	0.60 (0.088)	0.63 (0.089)

**Table 6:** Results for different workflows of random forests for hospital readmissions data, with standard deviations over n = 10 runs.

RF Workflow	ROC AUC	Sensitivity	Specificity	Accuracy
No Transformation	0.60 (0.047)	0.58(0.042)	0.57 (0.049)	0.57 (0.046)
No Transformation, SMOTE	0.52 (0.053)	0.46 (0.052)	0.75 (0.055)	0.70 (0.055)
No Transformation, ROSE	0.5 (0)	0 (0)	1(0)	0.827 (0)
PCA	0.56(0.051)	0.50 (0.066)	0.63 (0.068)	0.61 (0.068)
PCA, SMOTE	0.57 (0.053)	0.62(0.051)	0.60 (0.058)	0.60(0.056)
PCA, ROSE	0.53 (0.072)	0.54 (0.071)	0.56 (0.076)	0.56 (0.075)
Mapper, No Transformations	0.49 (0.078)	0.46 (0.084)	0.60 (0.081)	0.58 (0.082)
Mapper, No Transformations, SMOTE	0.55 (0.087)	0.58 (0.075)	0.54 (0.082)	0.55 (0.080)
Mapper, No Transformation, ROSE	0.51 (0.093)	0.54(0.116)	0.51 (0.143)	0.52 (0.137)
Mapper, Node PCA	0.57 (0.069)	0.62 (0.076)	0.62 (0.086)	0.62 (0.083)
Mapper, Node PCA, SMOTE	0.57 (0.084)	0.46 (0.095)	0.71 (0.091)	0.67 (0.092)
Mapper, Node PCA, ROSE	0.64 (0.110)	0.65 (0.099)	0.61 (0.091)	0.62 (0.097)

**Table 7:** Results for different workflows of Adaboost classifiers, with standard deviations over n = 10 runs.

AdaBoost workflow	ROC AUC	Sensitivity	Specificity	Accuracy
No Transformation	0.50 (0.043)	0.54 (0.058)	0.49 (0.049)	0.50 (0.051)
No Transformation, SMOTE	0.62 (0.056)	0.65 (0.072)	0.53 (0.070)	0.55 (0.071)
No Transformation, ROSE	0.5(0)	0 (0)	1 (0)	0.827 (0)
PCA	0.48 (0.038)	0.54 (0.044)	0.53 (0.049)	0.53 (0.048)
PCA, SMOTE	0.53 (0.051)	0.50 (0.053)	0.58 (0.057)	0.57 (0.056)
PCA, ROSE	0.69 (0.073)	0.46 (0.078)	0.74 (0.064)	0.69 (0.068)
Mapper, No Transformations	0.56 (0.079)	0.49 (0.082)	0.75 (0.086)	0.71 (0.085)
Mapper, No Transformations, SMOTE	0.63 (0.083)	0.73 (0.077)	0.63 (0.083)	0.65 (0.082)
Mapper, No Transformation, ROSE	0.54 (0.098)	0.42 (0.131)	0.67 (0.110)	0.63(0.119)
Mapper, Node PCA	0.63 (0.066)	0.69 (0.075)	0.58 (0.082)	0.60 (0.081)
Mapper, Node PCA, SMOTE	0.58 (0.088)	0.65 (0.084)	0.54 (0.091)	0.56 (0.089)
Mapper, Node PCA, ROSE	0.44 (0.141)	0.58 (0.109)	0.51 (0.092)	0.52(0.095)

**Fig 2:**
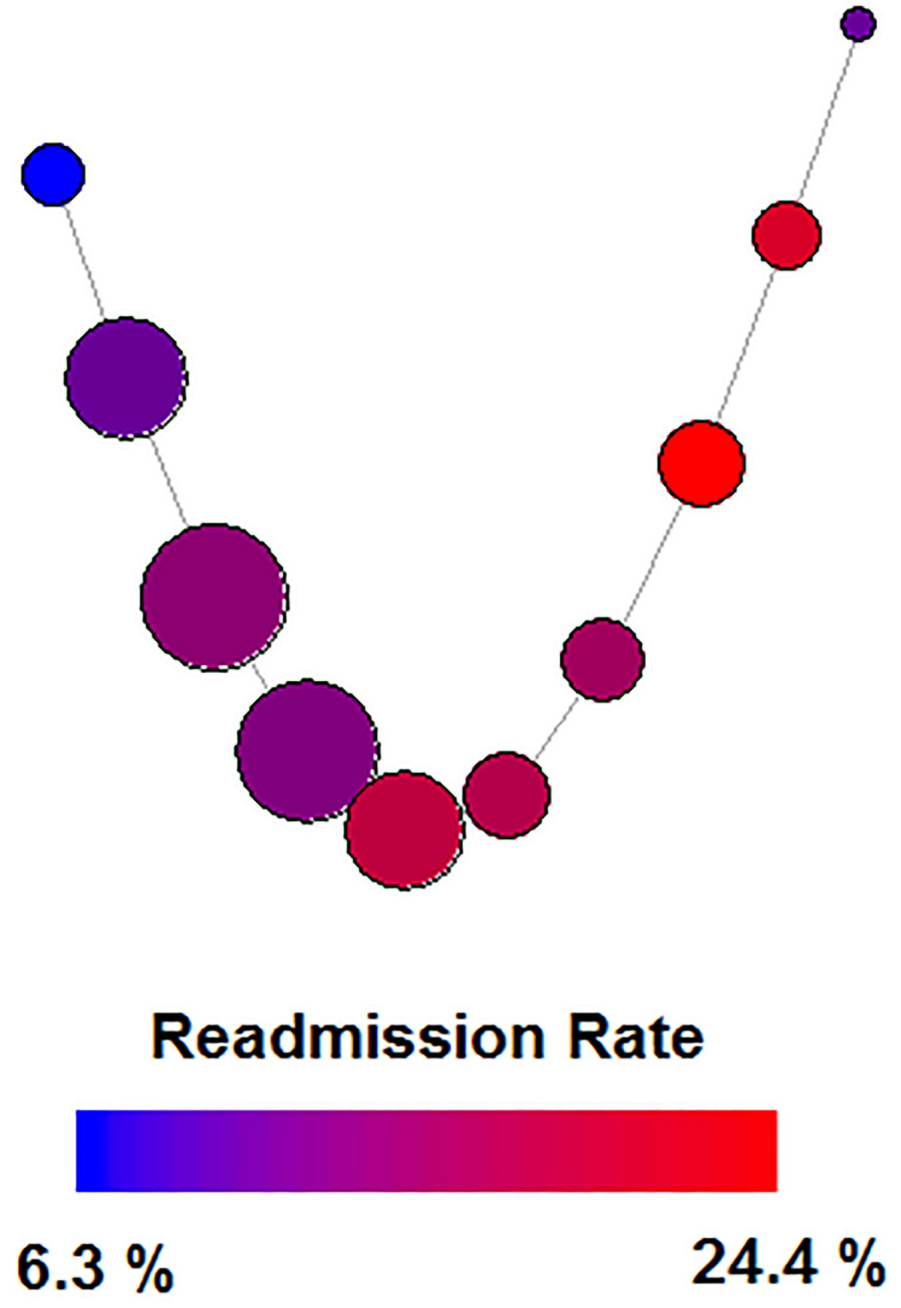
Typical Mapper graph generated from hospital readmissions data. The nodes are colored showing level of readmissions, and larger node size indicates a higher number of patients in that node.

The Mapper graphs were tuned using the first principal component of the entire dataset as the filtration function, a typical graph is shown in Fig 2. Based off of a grid search, a bin overlap of 40-50% yielded roughly the same results, with 10 intervals as the clustering parameter. Each run produced 5-10 nodes, with readmission ranging from 5%-30%.

This dataset catalyzed the use of the Mapper graph in the ML workflow. Models trained on the entire dataset do not perform well, and we were aiming to build a workflow that improved upon the LACE score. LACE is a combination of Length of previous hospital stays, Acuity of admission (emergency/not emergencey), the Charlson Comorbidity, and number of prior Emergency Department visits, and is the most used tool to predict readmissions risk. However, our population has a huge majority of “high” risk patients, and logistic regression trained on lace results in a ROC AUC of 0.59, with the optimal sensitivity at 0.54, which only correctly predicts slightly more than half of all readmissions. Applying Mapper in to our workflow resulted in large performance increases over the LACE model.

On the Logistic Regression, the Mapper algorithm with PCA computed for individual nodes and SMOTE sampling outperforms all other workflows, with Mapper/No Transformation (NT) and NT + SMOTE also showing higher performance than others. Sensitivity is of particular interest in this problem, in order to identify as many high risk patients as possible and target them with additional resources.

The SVM classifier performed the best out of the out-of-the-box models with no sampling or other transformations, however the Sensitivity was significantly increased by the models using Mapper, Node PCA, and SMOTE/ROSE sampling. None of the models using Mapper in conjunction with Random Forests showed large improvement over training the Random Forests over the entire training set. It should be noted that the models running Random Forests with ROSE sampling in the Caret Package didn’t converge, and voted “no-readmission” for every point in the test set. This issue also occured when using Adaboost classifiers with ROSE sampling in Table 7.

Adaboost models were improved both by sampling and in two of the Mapper workflows. One case to note is the PCA+ROSE combination, which features a “high” AUC but low Sensitivity. In this case we would throw out the model in favor of the Mapper, no transformation, SMOTE workflow which correctly identifies almost 3/4 of the readmitted patients. One reason AdaBoost models might perform better with the Mapper algorithm is that AdaBoost is often used on smaller datasets, which the Mapper workflow naturally creates.

Compared to the out of the box models trained using caret, workflows utilizing Mapper tend to have a higher variance between runs. This can be explained by additional variance introduced by assigning the testing points to different nodes of the Mapper graph. Since each run has a different testing set, different node models will predict different numbers of testing points. Additionally, variance is introduced by using SMOTE or ROSE sampling methods, since each run creates a new set of synthetic samples. Methods using ROSE Sampling and Mapper had the highest spread in metrics.

#### 4.2.1 Approval

The Washington University Institutional Review Board approved the use of this data as a retrospective study. All HIPPA identifying information has been removed.

### 4.2 Additional testing: German credit data

**Fig 3:**
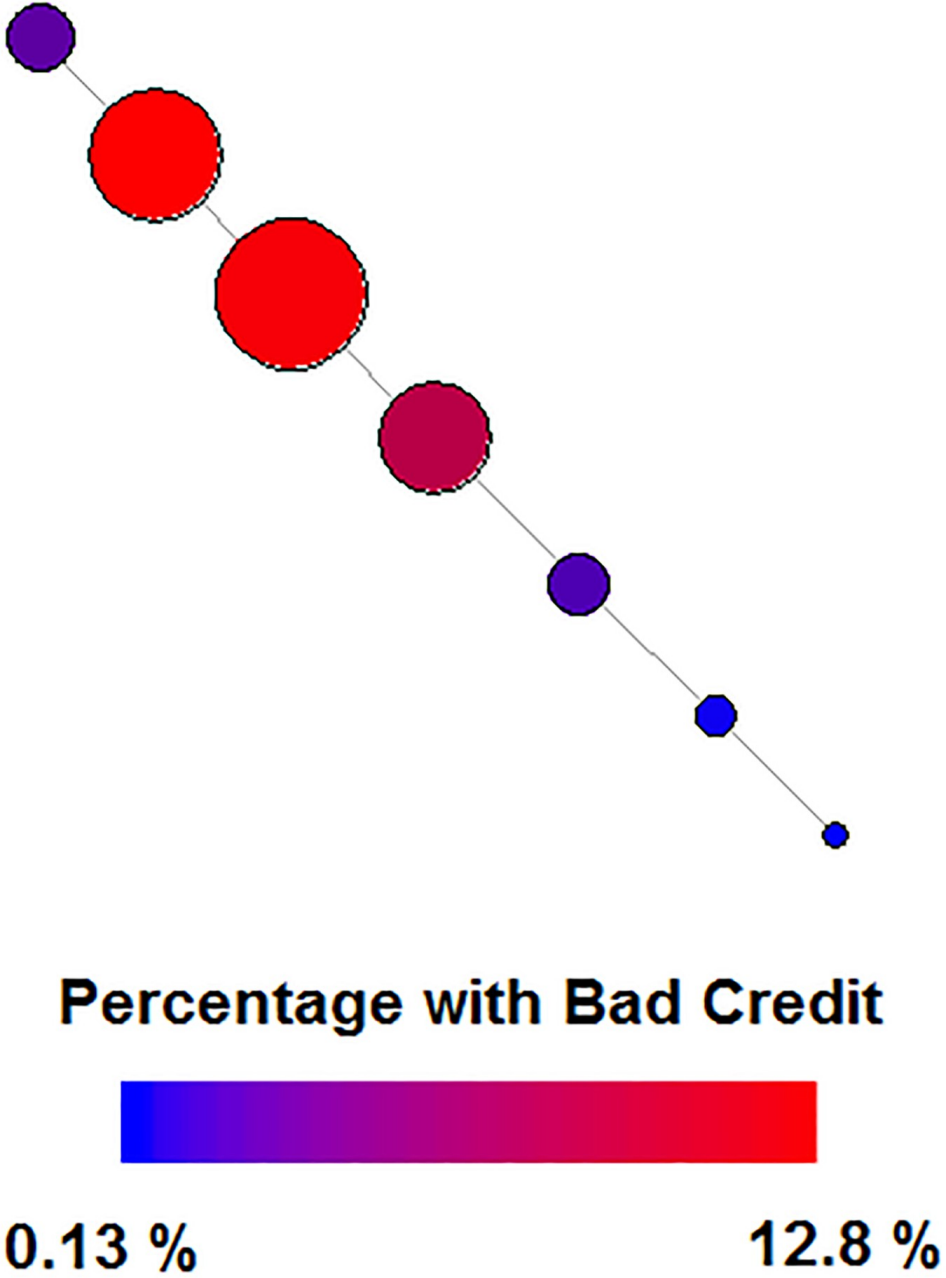
Typical Mapper Graph generated from first principal component of German Credit Data. Nodes are colored to show the levels of bad credit, and sized by number of data points.

For replication and additional testing, we built workflows on German Credit data available through UCI’s machine learning data repository [41]. The data features 20 features, which are described in Table 8. We chose this dataset because it has a mix of categorical and continuous features, and a similar class imbalance to the Hospital Readmissions data. The selected Mapper parameters were 10 intervals, 30% overlap, and 15 bins when clustering. A typical Mapper graph created 7 nodes, with 50-400 points per node, and is shown in Fig 3. The goal of these workflows is to classify applicants into “good” or “bad” credit risk.

**Table 8:** Descriptive table of German Credit Dataset from UCI Repository, monetary values in units of Deutsch Marks.

	Total Cohort (N = 1000)	Number with Bad Credit (Total = 300)
Checking Status <0	274	135
Checking Status 0-200	269	105
Checking Status >200	63	14
Checking Status no account	394	46
Credit History—no history	40	25
Credit History—all paid	49	28
Credit History—existing, paid	530	169
Credit History—Delayed Previously	60	28
Credit History—critical/other debt	293	50
Buying New Car	234	89
Buying Used Car	103	17
Buying furniture/equipment	181	58
Buying Radio/TV	280	62
No Savings	183	32
Savings < 100	603	217
Savings > 100	214	51
Rent Housing	179	70
Own Housing	713	186
Free Housing	64	44
Unemployed/unskilled non resident	22	7
Unskilled Resident	200	56
Skilled	630	186
Highly Skilled/Self Employed/Management	148	51

In the numerical comparisons, we encountered convergence issues with the AdaBoost algorithm and ROSE sampling scheme, so we do not report experiments featuring those MLMs.

On the credit data, we also experimented with adding the weighted interactions between two models. The results of the comparisons are posted in Tables 9–11. The weights were either equal, or weighted towards the best ROC AUC. If a testing point was assigned to nodes *i* and *j*, we used the weights:

$$C_{i}=\frac{AUC_{i}}{AUC_{i}+AUC_{j}}$$ (30)

$$C_{j}=\frac{AuC_{j}}{AUC_{i}+AUC_{j}}$$ (31)

where *AUC*_*i*_ and *AUC*_*j*_ are the average of the ROC AUC’s computed on the holdout sets during cross validation.

**Table 9:** Results for different workflows of logistic regression classifiers on German Credit Data, with standard deviations over n = 10 runs.

Model	ROC AUC	Sensitivity	Specificity	Accuracy
No Transformation	0.76 (0.043)	0.71 (0.033)	0.73 (0.039)	0.72 (0.038)
No Transformation, SMOTE	0.76 (0.027)	0.72 (0.046)	0.7 (0.041)	0.71 (0.043)
PCA	0.76 (0.031)	0.71 (0.041)	0.71 (0.035)	0.71 (0.037)
PCA, SMOTE	0.76 (0.049)	0.7 (0.046)	0.74 (0.048)	0.73 (0.048)
Mapper, 1 model	0.68 (0.054)	0.66 (0.063)	0.69 (0.057)	0.68 (0.059)
Mapper, SMOTE, 1 model	0.69 (0.068)	0.66 (0.060)	0.72 (0.061)	0.70 (0.061)
Mapper, 2 models, equal weight	0.73 (0.072)	0.71 (0.065)	0.69 (0.068)	0.70 (0.067)
Mapper, SMOTE 2 models, equal weight	0.72 (0.079)	0.71 (0.073)	0.7 (0.066)	0.70 (0.068)
Mapper, 2 models, AUC weight	.72 (0.075)	0.71 (0.068)	0.71 (0.063)	0.71 (0.064)
Mapper, SMOTE 2 models, AUC weight	0.72 (0.078)	0.7 (0.077)	0.67 (0.063)	0.68 (0.067)
Mapper, Node PCA, 1 model	0.74 (0.056)	0.73 (0.063)	0.67 (0.061)	0.69 (0.061)
Mapper, Node PCA, SMOTE, 1 model	0.71 (0.067)	0.69 (0.054)	0.68 (0.055)	0.683 (0.055)
Mapper, Node PCA, 2 models, equal weight	0.74 (0.057)	0.78 (0.063)	0.67 (0.069)	0.703 (0.068)
Mapper, Node PCA, SMOTE 2 models, equal weight	0.72 (0.073)	0.7 (0.076)	0.66 (0.067)	0.67 (0.069)
Mapper, Node PCA, 2 models, AUC weight	0.74 (0.072)	0.75 (0.059)	0.67 (0.063)	0.69 (0.062)
Mapper, Node PCA, SMOTE 2 models, AUC weight	0.74 (0.079)	0.7 (0.062)	0.69 (0.071)	0.69 (0.067)

**Table 10:** Results for different workflows of SVM classifiers on German Credit Data, with standard deviations over n = 10 runs.

Model	ROC AUC	Sensitivity	Specificity	Accuracy
No Transformation	0.77 (0.023)	0.74 (0.035)	0.71 (0.027)	0.72(0.029)
No Transformation, SMOTE	0.71 (0.038)	0.71 (0.049)	0.67 (0.042)	0.68 (0.044)
PCA	0.76 (0.028)	0.68 (0.051)	0.72 (0.038)	0.71 (0.041)
PCA, SMOTE	0.76 (0.036)	0.69 (0.031)	0.74 (0.043)	0.73 (0.040)
Mapper, 1 model	0.53 (0.051)	0.54 (0.055)	0.56 (0.047)	0.55(0.049)
Mapper, SMOTE, 1 model	0.54 (0.066)	0.57 (0.048)	0.55 (0.068)	0.56 (0.062)
Mapper, 2 models equal weight	0.52 (0.062)	0.54 (0.068)	0.56 (0.061)	0.55 (0.063)
Mapper, SMOTE 2 models—equal weight	0.54 (0.070)	0.54 (0.059)	0.59 (0.061)	0.58 (0.061)
Mapper, 2 models—AUC weight	.52 (0.064)	0.55 (0.054)	0.56 (0.072)	0.56 (0.065)
Mapper, SMOTE 2 models—AUC weight	0.53 (0.077)	0.59 (0.079)	0.52 (0.071)	0.54 (0.074)
Mapper, Node PCA, 1 model	0.72 (0.041)	0.73 (0.066)	0.67 (0.059)	0.69 (0.061)
Mapper, Node PCA, SMOTE, 1 model	0.76 (0.058)	0.75 (0.052)	0.67 (0.047)	0.69 (0.048)
Mapper, Node PCA, 2 models equal weight	0.75 (0.067)	0.7 (0.075)	0.69 (0.071)	0.7 (0.073)
Mapper, Node PCA, SMOTE 2 models, equal weight	0.76 (0.072)	0.72 (0.063)	0.71 (0.069)	0.71 (0.067)
Mapper, Node PCA, 2 models AUC weight	0.75 (0.056)	0.71 (0.059)	0.7 (0.067)	0.71 (0.064)
Mapper, Node PCA, SMOTE 2 models, AUC weight	0.76 (0.081)	0.73 (0.073)	0.71 (0.065)	0.72 (0.068)

**Table 11:** Results for different workflows of random forest classifiers on German Credit Data, with standard deviations over n = 10 runs.

Model	ROC AUC	Sensitivity	Specificity	Accuracy
No Transformation	0.75 (0.029)	0.72 (0.027)	0.71 (0.038)	0.71 (0.034)
No Transformation, SMOTE	0.76 (0.033)	0.7 (0.036)	0.73 (0.031)	0.72 (0.033)
PCA	0.75 (0.025)	0.69 (0.038)	0.71 (0.029)	0.7 (0.032)
PCA, SMOTE	0.75 (0.046)	0.71 (0.049)	0.72 (0.053)	0.72 (0.051)
Mapper, 1 model	0.7 (0.053)	0.75 (0.066)	0.62 (0.054)	0.66 (0.056)
Mapper, SMOTE, 1 model	0.71 (0.064)	0.7 (0.067)	0.67 (0.075)	0.68 (0.072)
Mapper, 2 models equal weight	0.71 (0.073)	0.75 (0.077)	0.65 (0.073)	0.68 (0.074)
Mapper, SMOTE 2 models—equal weight	0.69 (0.070)	0.68 (0.071)	0.67 (0.087)	0.68 (0.083)
Mapper, 2 models—AUC weight	.71 (0.061)	0.77 (0.078)	0.64 (0.074)	0.68 (0.075)
Mapper, SMOTE 2 models—AUC weight	0.69 (0.086)	0.67 (0.062)	0.7 (0.073)	0.68 (0.070)
Mapper, Node PCA, 1 model	0.67 (0.045)	0.64 (0.067)	0.65 (0.071)	0.65 (0.069)
Mapper, Node PCA, SMOTE, 1 model	0.67 (0.051)	0.65 (0.062)	0.66 (0.066)	0.66 (0.064)
Mapper, Node PCA, 2 models equal weight	0.66 (0.068)	0.67 (0.059)	0.64 (0.064)	0.65 (0.062)
Mapper, Node PCA, SMOTE 2 models, equal weight	0.67 (0.078)	0.67 (0.071)	0.67 (0.068)	0.67 (0.070)
Mapper, Node PCA, 2 models AUC weight	0.67 (0.075)	0.64 (0.060)	0.68 (0.058)	0.65 (0.059)
Mapper, Node PCA, SMOTE 2 models, AUC weight	0.67 (0.073)	0.68 (0.067)	0.68 (0.072)	0.68 (0.071)

An interesting property of this dataset is that training models with no transformations yields high ROC AUC (compared to models trained on the readmissions data) across every model, with roughly the same performance when PCA is applied across the whole dataset. The Mapper/Logistic Regression workflow produced lower ROC AUC’s when no transformations were used, but similar AUC’s when node PCA was applied, and slightly lower specificity.

Random forest classifiers using the Mapper workflow performed worse in AUC than training a random forest on the original dataset. Most of the loss in performance is due to much lower specificity across the board. When applying node PCA to random forests, the performance decreased, unlike the Logistic Regression case.

SVM classifiers with Mapper also performed poorly across the board when no node transformations are applied. However, applying PCA to Mapper nodes significantly outperformed Mapper workflows with no transformations, and had similar results to workflows trained on the entire dataset.

When a weighted combination of two models was used instead of one model (more than one *C*_*i*_(**x**) > 0), the results were either the same or slightly improved. One reason for the same results is that models tend to be similar when trained with nodes that overlap with each other, while models from nodes on opposite ends of the Mapper graph are different. This means models give roughly the same probability, and therefore the weighted combination will be roughly the same as just using the closest model. More experimentation and analysis should be done to tune these weights.

## 5 Discussion

In summary, we presented the machine learning workflow as a composition of machine learning morphisms, starting from the original data space X, and ending with task completion. We presented several properties of these morphisms, including when groups of MLMs form a vector space. Then we presented a workflow using the TDA Mapper algorithm as an MLM to split **X** into overlapping subsets and train models on each subset. We discussed implications of this workflow and presented results from two applications on real data. In the case of Hospital Readmissions we found several models using the Mapper algorithm that yielded increased performance. In the case of the German Credit data, models using Mapper and node PCA performed similarly to workflows trained without Mapper. However, the closeness of some of the models suggests that further tuning may be all that is needed to improve performance.

Besides potential performance improvements, workflows utlizing Mapper provide the advantage of parallelization. On the German Credit Data, we showed several cases where models trained on smaller datasets and then pooled together performed just as well as models trained on the whole dataset. Once the data is split, each model is trained on a significantly smaller portion of the data. The training can be parallelized, which has the potential to significantly decrease training time on large datasets, or on models such as neural networks and SVM that scale nonlinearly with the number of data points.

Some limitations with the numerical experiments include limited data: each node is only assigned a few samples from the test set, which adds some uncertainty to the results. The bounds of uncertainty should be further analyzed, and these workflows should be analyzed by studying datasets with more samples.

One direction for future work is to examine the theoretical performance of the workflow utilizing TDA. TDA is a natural fit for this framework because it is intrinsically interested in the study of data spaces and the morphisms between those spaces.

Other directions of future work include studying the optimization of MLM based workflows given a choice of risk functions. When should parameters be optimized independently, and when should they be formulated as a joint optimization over a risk function? When the morphisms and risk functions are all continuous and differentiable functions, the workflow is similar or equivalent to a simple feed forward neural network, and we could leverage the research done on neural networks to jointly optimize large ensembles of models. Finally, vector spaces of MLMs could be leveraged to build and analyze ensembles.

In conclusion, the MLM framework allows us to concisely build multiple well organized workflows, from preprocessing to task completion, and provides a medium for performance analysis and optimization of all workflow parameters.

## 6 Appendix

[Proof of Property 1]

*Proof*. Given that they have the same output space, to show that $ML_{3}={ML}_{1}⊕ML_{2}$, it is sufficient to establish that the optimal parameters of $ML_{3}$ are the same as the optimal parameters of ${ML}_{1}$ and $ML_{2}$.

Let

$$p_{1}^{*}=\underset{p\in \Theta_{1}}{\text{arg}\;\text{min}}\;{\bar{R}}_{1}\left(p_{1};X,Y,F_{1}\left(\cdot ;p_{1}\right),P\left(p_{1}\right)\right)$$

and

$$p_{2}^{*}=\underset{p\in \Theta_{2}}{\text{arg}\;\text{min}}\;{\bar{R}}_{2}\left(p_{2};X,Y,F_{2}\left(\cdot ;p_{2}\right),P\left(p_{2}\right)\right)$$

be the optimal parameters of ${ML}_{1}$ and $ML_{2}$. Now find the optimal parameters of ${\bar{R}}_{3}$:

$$\underset{\left(p_{1},p_{2}\right)\in \Theta_{1}\times \Theta_{2}}{\text{arg}\;\text{min}}\;{\bar{R}}_{3}\left(\left(p_{1},p_{2}\right);X_{1}\cup X_{2},Y,F_{1}⊕F_{2},P\left(\left(p_{1},p_{2}\right)\right)\right)=$$ (32)

$$\underset{\left(p_{1},p_{2}\right)\in \Theta_{1}\times \Theta_{2}}{\text{arg}\;\text{min}}\;a{\bar{R}}_{1}+b{\bar{R}}_{2}=$$

$$\left(\underset{p_{1}\in \Theta_{1}}{\text{arg}\;\text{min}}\;{\bar{R}}_{1},\underset{p_{2}\in \Theta_{2}}{\text{arg}\;\text{min}}\;{\bar{R}}_{2}\right)=\left(p_{1}^{*},p_{2}^{*}\right)$$

Since $\text{min}\left(a{\bar{R}}_{1}+b{\bar{R}}_{2}\right)=a\;\text{min}\left({\bar{R}}_{1}\right)+b\;\text{min}\left({\bar{R}}_{2}\right)$, therefore we can optimize ${\bar{R}}_{1}$ and ${\bar{R}}_{2}$ independently in ${\bar{R}}_{3}$.

[Decomposition of Linear Regression with Orthogonal Training Matrix] The risk function for linear least squares regression is:

$${\bar{R}}_{3}=∥Y-{Xp∥}_{2}^{2}={\left(Y-Xp\right)}^{T}\left(Y-Xp\right)=$$ (33)

which expands to:

$$Y^{T}Y-\left[p_{1}^{T},p_{2}^{T}\right]{\left[X_{1}X_{2}\right]}^{T}Y-Y^{T}\left[X_{1}X_{2}\right][\begin{array}{c} p_{1}\\ p_{2} \end{array}]+\left[p_{1}^{T},p_{2}^{T}\right]{\left[X_{1}X_{2}\right]}^{T}\left[X_{1}X_{2}\right][\begin{array}{c} p_{1}\\ p_{2} \end{array}]=$$ (34)

continuing the matrix multiplication:

$$Y^{T}Y-p_{1}^{T}X_{1}^{T}Y-Y^{T}X_{1}p_{1}-p_{2}^{T}X_{2}^{T}Y-Y^{T}X_{2}p_{2}+\left(p_{1}^{T}X_{1}^{T}+p_{2}^{T}X_{2}^{T}\right)\left(X_{1}p_{1}+X_{2}p_{2}\right)=$$ (35)

cancelling out the cross terms:

$$Y^{T}Y-p_{1}^{T}X_{1}^{T}Y-Y^{T}X_{1}p_{1}-p_{2}^{T}X_{2}^{T}Y-Y^{T}Y+Y^{T}Y-Y^{T}X_{2}p_{2}+p_{1}^{T}X_{1}^{T}X_{1}p_{1}+p_{2}^{T}X_{2}^{T}X_{2}p_{2}=$$ (36)

finally, collecting the terms with **X**_1_ and **X**_2_ into a sum of norms:

$$-\frac{1}{2}{∥Y∥}_{2}^{2}+∥Y-X_{1}p_{1}{∥}_{2}^{2}-\frac{1}{2}{∥Y∥}_{2}^{2}+∥Y-X_{2}p_{2}{∥}_{2}^{2}={\bar{R}}_{1}+{\bar{R}}_{2}$$ (37)

## References

[1] Vapnik V. The nature of statistical learning theory. Springer science & business media; 2013.

[2] Abadi M, Barham P, Chen J, Chen Z, Davis A, Dean J, et al Tensorflow: a system for large-scale machine learning. In: OSDI. vol. 16; 2016 p. 265–283.

[3] Ball D. Induction by a Hilbert hypercube representation. Aston University; 1991.

[4] Singh G, Mémoli F, Carlsson GE. Topological methods for the analysis of high dimensional data sets and 3d object recognition. In: SPBG; 2007 p. 91–100.

[5] Miescke KJ, Liese F. Statistical Decision Theory: Estimation, Testing, and Selection.

[6] Pednault EP. Statistical learning theory. Citeseer; 1997.

[7] Vapnik VN. An overview of statistical learning theory. IEEE transactions on neural networks. 1999;10(5):988–999. 10.1109/72.788640 18252602

[8] Xuegong Z. Introduction to statistical learning theory and support vector machines. Acta Automatica Sinica. 2000;26(1):32–42.

[9] Nasrabadi NM. Pattern recognition and machine learning. Journal of electronic imaging. 2007;16(4):049901 10.1117/1.2819119

[10] Vapnik V. Principles of risk minimization for learning theory. In: Advances in neural information processing systems; 1992 p. 831–838.

[11] Olson RS, Moore JH. TPOT: A Tree-based Pipeline Optimization Tool for Automating Machine Learning. Springer; 2018 p. 163–173.

[12] Feurer M, Klein A, Eggensperger K, Springenberg JT, Blum M, Hutter F. Auto-sklearn: Efficient and Robust Automated Machine Learning. Springer; 2018 p. 123–143.

[13] Kotthoff L, Thornton C, Hoos HH, Hutter F, Leyton-Brown K. Auto-WEKA: Automatic model selection and hyperparameter optimization in WEKA. Springer; 2018 p. 89–103.

[14] Witten IH, Frank E, Hall MA, Pal CJ. Data Mining: Practical machine learning tools and techniques. Morgan Kaufmann; 2016.

[15] Chawla NV, Bowyer KW, Hall LO, Kegelmeyer WP. SMOTE: synthetic minority over-sampling technique. Journal of artificial intelligence research. 2002;16:321–357. 10.1613/jair.953

[16] Lunardon N, Menardi G, Torelli N. ROSE: A Package for Binary Imbalanced Learning. R journal. 2014;6(1). 10.32614/RJ-2014-008

[17] Raiber F, Kurland O. Kullback-Leibler Divergence Revisited. In: Proceedings of the ACM SIGIR International Conference on Theory of Information Retrieval. ICTIR’17. New York, NY, USA: ACM; 2017. p. 117–124. Available from: http://doi.acm.org/10.1145/3121050.3121062.

[18] Rish I, et al. An empirical study of the naive Bayes classifier. In: IJCAI 2001 workshop on empirical methods in artificial intelligence. vol. 3. IBM New York; 2001. p. 41–46.

[19] Barandiaran, Iñigo. The random subspace method for constructing decision forests. IEEE Trans. Pattern Anal. Mach. Intell; volume 20; number 8; 1-22, 1998.

[20] Cerda P, Varoquaux G, Kégl B. Similarity encoding for learning with dirty categorical variables. Machine Learning. 2018; p. 1–18.

[21] Patel N, Upadhyay S. Study of various decision tree pruning methods with their empirical comparison in WEKA. International journal of computer applications. 2012;60(12). 10.5120/9744-4304

[22] Rojas R. AdaBoost and the super bowl of classifiers a tutorial introduction to adaptive boosting. Freie University, Berlin, Tech Rep. 2009.

[23] Mehrotra K, Mohan CK, Ranka S. Elements of artificial neural networks. MIT press; 1997.

[24] Data Preparation and Feature Engineering in ML; 2018. Available from: https://developers.google.com/machine-learning/data-prep/.

[25] Fawcett T. An introduction to ROC analysis. Pattern recognition letters. 2006;27(8):861–874. 10.1016/j.patrec.2005.10.010

[26] Akaike H. Information theory and an extension of the maximum likelihood principle In: Selected papers of hirotugu akaike. Springer; 1998 p. 199–213.

[27] Wasserman L. Topological data analysis. Annual Review of Statistics and Its Application. 2018;5:501–532. 10.1146/annurev-statistics-031017-100045

[28] Bubenik P. Statistical topological data analysis using persistence landscapes. The Journal of Machine Learning Research. 2015;16(1):77–102.

[29] Gamble J, Heo G. Exploring uses of persistent homology for statistical analysis of landmark-based shape data. Journal of Multivariate Analysis. 2010;101(9):2184–2199. 10.1016/j.jmva.2010.04.016

[30] Bendich P, Marron JS, Miller E, Pieloch A, Skwerer S. Persistent homology analysis of brain artery trees. The annals of applied statistics. 2016;10(1):198 10.1214/15-AOAS886 27642379 PMC5026243

[31] Gholizadeh S, Seyeditabari A, Zadrozny W. Topological Signature of 19th Century Novelists: Persistence Homology in Context-Free Text Mining. 2018.

[32] Duponchel L. Exploring hyperspectral imaging data sets with topological data analysis. Analytica chimica acta. 2018;1000:123–131. 10.1016/j.aca.2017.11.029 29289301

[33] Nicolau M, Levine AJ, Carlsson G. Topology based data analysis identifies a subgroup of breast cancers with a unique mutational profile and excellent survival. Proceedings of the National Academy of Sciences. 2011; p. 201102826. 10.1073/pnas.1102826108 PMC3084136 21482760

[34] Coudriau M, Lahmadi A, François J. Topological analysis and visualisation of network monitoring data: Darknet case study. In: Information Forensics and Security (WIFS), 2016 IEEE International Workshop on. IEEE; 2016. p. 1–6.

[35] Carriere M, Michel B, Oudot S. Statistical analysis and parameter selection for Mapper. The Journal of Machine Learning Research. 2018;19(1):478–516.

[36] Epstein C, Carlsson G, Edelsbrunner H. Topological data analysis. Inverse Problems. 2011;27(12):120201 10.1088/0266-5611/27/12/120201

[37] Gao X, Xiao B, Tao D, Li X. A survey of graph edit distance. Pattern Analysis and applications. 2010;13(1):113–129. 10.1007/s10044-008-0141-y

[38] Menardi G, Torelli N. Training and assessing classification rules with imbalanced data. Data Mining and Knowledge Discovery. 2014;28(1):92–122. 10.1007/s10618-012-0295-5

[39] Rahman A, Verma B. Novel layered clustering-based approach for generating ensemble of classifiers. IEEE Transactions on Neural Networks. 2011;22(5):781–792. 10.1109/TNN.2011.2118765 21486714

[40] Kuhn M. The caret Package; 2009.

[41] Dheeru D, Karra Taniskidou E. UCI Machine Learning Repository; 2017. Available from: http://archive.ics.uci.edu/ml.

