# Correction: Designing machine learning workflows with an application to topological data analysis

**DOI:** 10.1371/journal.pone.0229821

**Published:** 2020-02-26

**Authors:** Eric Cawi, Patricio S La Rosa, Arye Nehorai

In the Author Contributions, Patricio S. La Rosa should be listed as one of the persons responsible for Formal Analysis, Methodology, and Writing–Review and Editing.
